# The Effect of Using a Metal Tube on Laser Welding of the Battery Case and the Tab for Lithium-Ion Battery

**DOI:** 10.3390/ma13194460

**Published:** 2020-10-08

**Authors:** Lanh Ngoc Trinh, Dongkyoung Lee

**Affiliations:** 1 Department of Future Convergence Engineering, Kongju National University, 1223-24 Cheonandaero, Seobuk-gu, Cheonan 31080, Korea; trinhngoclanh199633@gmail.com; 2 Department of Mechanical and Automotive Engineering, Kongju National University, 1223-24 Cheonandaero, Seobuk-gu, Cheonan 31080, Korea

**Keywords:** metal tube, laser welding, IMCs, tensile strength, electrical resistance, lithium-ion battery

## Abstract

Given the drawbacks of the conventional welding methods in joining the battery case and tab in the lithium-ion battery, the laser welding technique using the metal tube has been introduced for the weld. The metal tube is supposed to contribute a positive effect including protection to the outside structure by blocking the injection of the spatters, and minimization of the contact gap between the battery case and table. However, the use of the metal tube is believed to cause the plume trapped inside and affect the intensity distribution of the laser gaussian beam. Through the observation and analysis in this study, both advantages and disadvantages of the application of the metal tube on the weld have been analyzed. The use of the metal tube prevents the ejection of the spatter to the outside of the welding zone, as well as minimize the air gap between the battery case and tab in the lap joint weld is also minimized. On the other hand, the trapped plume inside the metal tube and the reduction of the energy of the laser beam have been considered to cause significant changes in the morphology, mechanical, and electrical properties of the weld.

## 1. Introduction

**Figure 1:**
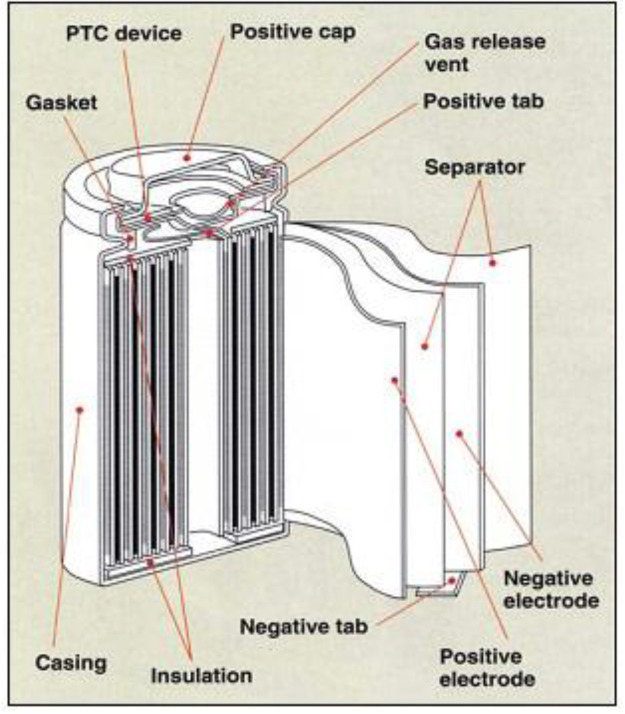
Cross-section of a lithium-ion battery cylindrical cell.

Nowadays, the use of lithium-ion batteries is increasing rapidly due to its multiple advantageous features. Long lifespan, environmental friendliness, and low-cost are practical requirements for the energy storage system in our modern industry. Having met these requirements, the rechargeable lithium-ion battery has been employed as the power supply or energy storage system in various industries, especially in automotive applications [1,2,3,4,5,6]. The lithium-ion battery has outstanding characterizations such as high energy intensity, high power density, low self-discharge rate, and a wide temperature range of operation in comparison with conventional batteries [7]. The design of a lithium-ion battery cell is based on its applications. The typical battery cell designs include button cell, prismatic cell, cylindrical cell, and pouch cell. Among the different cell designs, the cylindrical cell is easy to be manufactured in comparison with the others. Additionally, the given cell design attributes with high specific energy, good mechanical stability, and long cycle life [8,9,10,11] As a result, the cylindrical cell is the most common cell design used for primary and secondary batteries. The major physical components of a cylindrical cell battery are jellyroll, current collectors, and safety devices as shown in Figure 1. A jellyroll consists of a positive electrode, a negative electrode, and separators, which are prepared in sheets. The jellyroll comprises a separator located between the positive and negative electrode sheets. In the cylindrical cell, the current collectors are the positive and negative terminals used for electron transmission. The safety devices include battery case, insulation plates, exhaust gas hole, anti-explosive valve which assist and protect the energetic materials and electrolyte inside the battery cell.

**Figure 2:**
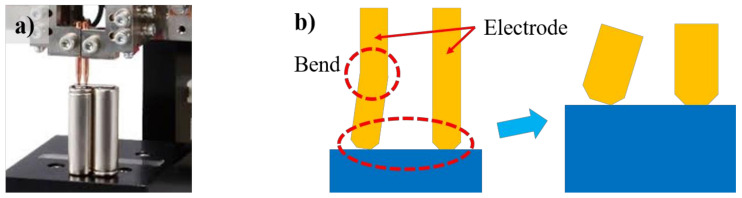
(**a**) Resistance spot welding, (**b**) Electrode bending due to high temperature.

During the battery manufacturing process, there are several connections that must be conducted with welding. Above all, the weld between battery case and tab is considered as the most challenging for the manufacturers due to the dissimilar materials, their thicknesses, and low electrical resistance requirement. The tab is usually manufactured with copper or aluminum as its high electrical conductivity as well as low weight per unit volume ratio. Meanwhile, the battery case is required to have high strength as well as high corrosion resistance so that it can prevent the central part of the battery from the deformation caused by an external force. Steel is one of the most suitable materials which can meet the requirements. Moreover, the battery case is commonly manufactured with a nickel plating layer on the surface to improve the joint and increase the electrical conductivity of the joint [12]. In joining the battery case and tab, the typical challenge is the difference in chemical and physical properties of the material of the battery case and the table. Steel has a melting temperature of around 1425–1540 °C, while the melting point of aluminum is 660 °C. This makes it difficult to weld these materials using traditional fusion welding technologies. Moreover, the low solubility of the dissimilar materials causes the formation of intermetallic compounds (IMCs) which negatively affects the mechanical properties of the joint. The IMCs are believed to cause the formation of several weld defects such as pores and cracks which causes the low strength of the weld [13,14,15,16,17]. Recently, many manufacturers have used the resistance spot welding method to weld the tab and battery case during battery manufacturing. However, the resistance spot welding can only weld metals with the thickness range from 5 to 50 inches. Besides, the resistance spot welding also results in the deep weld and narrow weld nugget [18]. Besides, a practical issue has been detected during the welding process. After many times of operation, the electrodes are bent due to high temperature as well as pressure from the upper system, shown in Figure 2.

As the electrodes are bent, the electrodes improperly contact the workpiece, this might cause the non-uniform welds. Therefore, the need of improving the welding method or finding a substitute welding technique is essential. Previously, several studies have investigated the joint of these dissimilar materials using different methods. A solid-state welding method was first thought to have the potential for joining the dissimilar materials. Taban et al. [19] have attempted to weld the dissimilar metals of 6061-T6 aluminum and AISI 1018 steel using friction welding technique. The result showed that the strength of the weld was in the interval of 170–250 MPa. Besides, the thickness of the intermetallic compounds layer at the interface of the joint was measured at about 350 nm. Its composition was suggested including FeAl (Fe-rich) and Fe_2_Al_5_ (Al-rich). However, one of the drawbacks of this welding technique was the relatively long welding cycle (time). Another solid-state welding technique, ultrasonic welding, has been attempted to weld these dissimilar metals. Patel et al. [20] have investigated the microstructural change and the mechanical performance of the dissimilar metal joint between aluminum alloy Al5754-O and the galvanized high-strength low-alloy (HSLA) steel welded by the ultrasonic spot-welding process. The analysis reported that the intermetallic compounds found at the interface were FeAl_3_ and Fe_2_Al_5_. The eutectic Al–Zn film was also found in the region. It was also reported that the failure during the tensile shear test could firstly take place between the eutectic Al–Zn film and the IMCs. When the load force increased, the joint was completely detached in the Al-containing region. However, this welding technique is only can be used on the designed joint (lap joint) as well as the technique requires custom tooling for every new project. Recently, an advanced technology, laser material processing, has been developing and applying in diverse industries, especially in lithium-ion battery manufacturing [21,22,23,24,25,26,27,28,29]. Moreover, the laser welding is well known for the non-contact process which provides various benefits for the weld such as low heat input, small heat-affected zone, fast processing, and low thermal deformation. Based on the analysis of the welding techniques for the dissimilar of aluminum and steel as well as the problem during the welding of tab and battery case using the conventional methods, the laser technology is introduced to improve the weld. Although laser welding technology has proposed various features to solve the above-mentioned problems of conventional welding, a critical request should be accomplished to get a good weld with laser welding technology. Since the laser welding is a non-contact process, the specimens should be fixed with a stable clamping system. During welding with overlap configuration, the metal evaporation with high pressure might lead to move and increase the gap between the upper and lower specimen if the fixture is not applied. Therefore, the objective of this study is to investigate the use of the metal tube which acts as a heat sink and reduces the air gap between the specimens (clamping effect) in the weld of the battery case and the table. Moreover, preventing the ejection of the spatter to the jellyroll during welding. However, the laser-induced plume trapped inside it during welding due to the use of the metal tube also needed to be considered. Therefore, it is needed to study the effect of the metal tube on the weld properties. In the present experiment, an investigation of the application of the metal tube in laser welding for the battery case and tab in the lithium-ion battery. Moreover, as the metal tube is placed over the tab and the battery case, the gap between them should be minimized. However, the metal tube might affect the laser beam transmission. Therefore, the investigation of using the metal tube in the process is needed. In other words, this experiment aims to investigate the effect of using a metal tube in laser welding of the battery case and tab in different laser powers. 

## 2. Materials and Methods

### 2.1. Materials

Due to the high electrical conductivity and lightweight characteristics, aluminum and copper have been used in many applications, especially in the electrical application. In lithium-ion batteries, the tab is usually made of copper and aluminum. Meanwhile, in the present experiment, the pure aluminum tab is used as the specimen for the weld. The dimension of the tab is given as 40 mm × 7 mm × 0.087 mm^3^ corresponding to length × width × thickness. In the cylindrical cell, the battery is manufactured to protect as well as prevent the central part of the battery from the external damages. Therefore, the material for the battery case must have sufficient strength and low-temperature deformation. Steel is considered as the most suitable material for the battery case as it is a well-known material for high strength as well as low thermal expansion coefficient. In this experiment, the battery case made of steel (chemical composition of 0.03% C, 0.003% Si, 0.23% Mn, 0.011% P, 0.008% S) plated with a thin nickel layer on the surface. The battery case is a commercial design with the thickness of 0.4 mm, and 73.5 mm in height. The outer diameter of the battery case is 21 mm. Moreover, a metal tube which the laser beam transfer through is also used. The inner diameter of the tube is 4.5 mm. The metal tube is made of aluminum and the length of the tube is 80 mm.

### 2.2. Experiment Procedure

**Figure 3:**
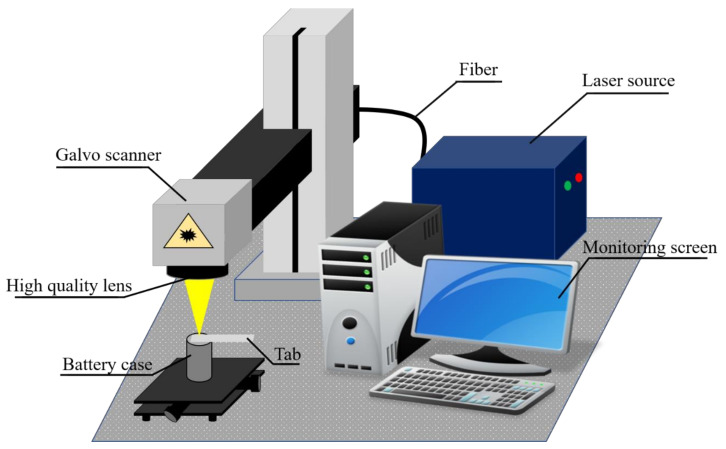
Experimental setup.

The experimental schematic for the weld of the battery case and the tab is shown in Figure 3. The laser source employed for the experiment is Ytterbium pulsed fiber laser (IPG-YLPM, IPG photonics, Southbridge, MA, USA). The feature of the laser source includes the maximum laser power of 20 W, the wavelength of 1064 nm, and the spot size (according to the definition of the 1/e^2^ of the beam width) of 30 μm In the current experiment, the laser power in the interval of 10–20 W is chosen to perform the weld. The additional laser parameters include pulse duration and repetition rate are set at 200 ns and 20 kHz, respectively. The pulsed laser has the capability to store and releases energy very rapidly. Therefore, we suppose that the use of the pulsed laser source would produce a weld with deep penetration and reduce the interaction time between the base materials. Thus, the formation of the brittle layer between the base materials can be reduced.

**Figure 4:**
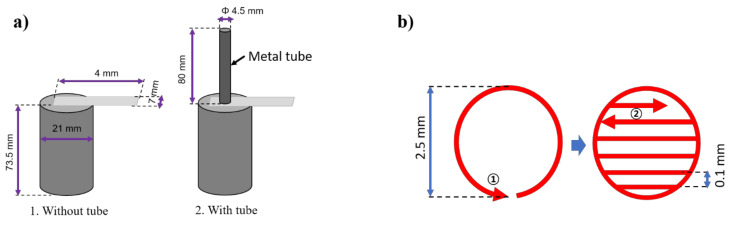
(**a**) Specimen setup, (**b**) Welding path.

The setup of the weld configurations, as well as dimensions for the specimens, are shown in Figure 4a. The experiment is conducted without the metal tube to compare with the experiment using the metal tube. In both cases, the tab is placed over the battery case and the weld is conducted in overlap configuration. The application of the metal tube derives from the need of the actual manufacturing issue. During manufacturing, the tab needs to be connected with the battery case and the joining process is conducted inside the jellyroll. Therefore, it is needed to protect the jellyroll during welding from the weld defects. Moreover, the gap between the battery case and the tab is found during welding which significantly weakens the weld joint. Thus, the metal tube is suggested to insert in the middle of the jellyroll to increase the contact between the tab and the battery case as well as protect the jellyroll from the spatter ejection. Therefore, the length of the metal tube should be at least longer than the battery case and the jelly roll (75 mm in length). In the present experiment, to get ease of observation and mechanical testing, the joint is performed outside of the battery case. In the weld with the metal tube, the tube is placed over the overlap weld, and the laser beam irradiates through the tube. The welding path is designed to be suitable for the movement of the laser irradiation inside the tube, which is exhibited in Figure 4b. The welding path consists of two parts: (1) circular line and (2) zigzag hatching lines. The advantages of this designed welding path are an increase in the contact area between the welded specimens as well as multiple options for the cross-section observation. 

In order to observed and analyze the experimental result, the Scanning Electron Microscope (SEM) with the model of VEGA3-SHB (TESCAN) (Brno, Brno-City, Czech Republic) is first used to observe the weld morphology. Next, the Energy Dispersive X-Ray (EDX, model: MIRA LMH (TESCAN)) (Brno, Brno-City, Czech Republic) is performed on the cross-section of the weld which is cut, grinding, and etching with 4% Nital solution. The mechanical properties of the weld are represented by the Vickers hardness and mechanical strength. In the Vickers hardness measurement, loads of 245.2 mN and 98.07 mN are applied to the vertical and horizontal measurements for 10 s on the cross-section of the weld, respectively. The mechanical strength of the weld is determined with the pulling test on the joints. An increasing tensile load is applied continuously to the welded specimens until they detach from each other, the maximum load that the weld undergoes is recorded. The four-wire method [30] is applied in this experiment for the electrical resistance measurement of the weld. A 4-point probe measurement system has been used to measure the electrical resistance of the weld, 2 probes are on the tab and the 2 other probes press on the battery case.

## 3. Results and Discussion

### 3.1. Effect of the Metal Tube on Gaussian Beam Distribution

It is interesting to note that the laser beam in the present experiment characterizes the Gaussian beam distribution. The calculation of the laser beam intensity according to the Gaussian beam has been clarified by William Steen [31]. In the present experiment, the use of the metal tube has affected the distribution of the laser beam (Gaussian beam) on the material. As mentioned above, the inner diameter of the metal tube is 4.5 mm, which means the intensity distribution of the laser beam (Gaussian beam) is limited by the area of the metal tube’s entrance. As a result, the power distribution of the laser beam transmitting into the metal tube should be integrated with respect to *r* in the limit from 0 to the radius of the metal tube (2.25 mm) as shown below:

(1) $$P_{metal\;tube}=\int_{0}^{2\pi }\int_{0}^{2.25}\frac{2P_{0}}{\pi {W_{R}}^{2}}\cdot e^{\frac{-2r^{2}}{{W_{R}}^{2}}}rdr\;d\theta$$

The result of the Equation (1) is:

(2) $$P_{0}\left(1-e^{\frac{-2.22,5^{2}}{{W_{R}}^{2}}}\right)$$

where *z* equals the length of the metal tube (80 mm). Consequently, the final result of the Equation (1) will be 0.95 *P*_0_. It means that 95% of *P*_0_ is transmitted through the metal tube. In other words, the use of the metal tube has affected power distribution. However, this influence of the metal tube on the power distribution of the laser beam seems not to cause significant change on the weld. Furthermore, besides the effect of the metal tube on the Gaussian beam propagation, the use of the metal tube causes the effect of plume trapped inside the metal tube. This plume effect also has a significant influence on the laser energy transmission inside the metal tube. Therefore, the effects of the loss of the laser energy caused by the trapped plume and power distribution on the weld characteristics are analyzed in the following sections.

### 3.2. Macrostructure

**Figure 5:**
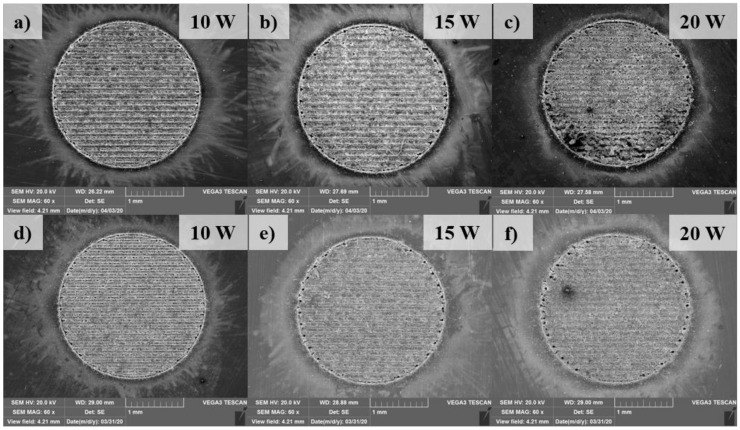
SEM images of the overview welding zone on the top surface. (**a**–**c**): Welding without the metal tube; (**d**–**f**): Welding with the metal tube.

**Figure 6:**
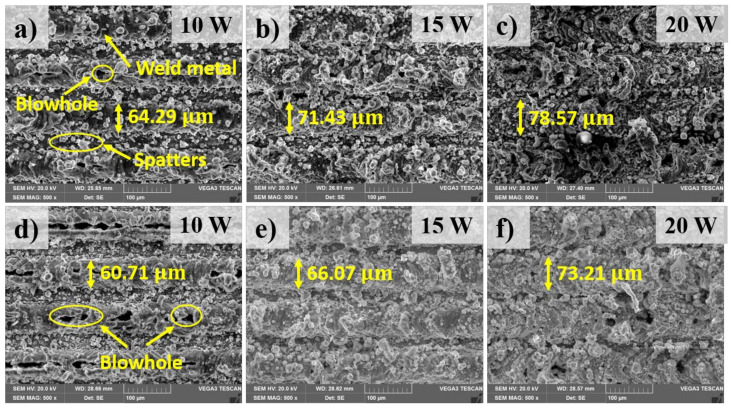
SEM images at the center of the welding zone on the top surface. (**a**–**c**): Welding without the metal tube; (**d**–**f**): Welding with the metal tube.

**Figure 7:**
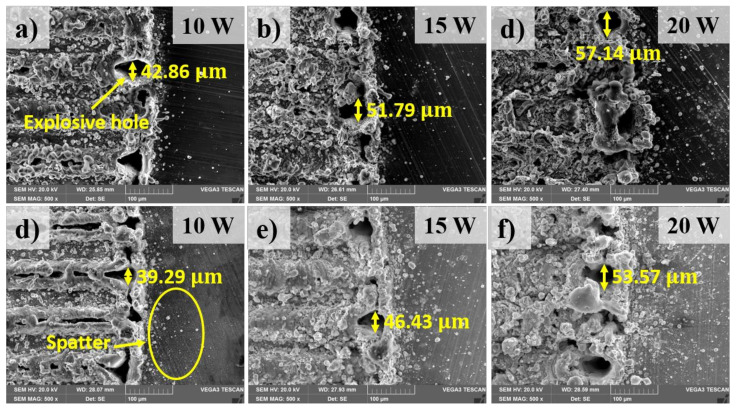
SEM images at the boundary of the welding zone on the top surface. (**a**–**c**): Welding without the metal tube; (**d**–**f**): Welding with the metal tube.

Figure 5 shows an overview of the top surfaces of the welding zone. The morphology of the weld is firstly analyzed with the observation on the top surface of the weld. Figure 6 shows the comparison of the top surface in the weld with and without the metal tube in different laser powers. This observation is taken in the middle of the welding zone. The top surface morphology shows a significant amount of the weld metal as well as several weld defects such as blowholes and spatters. The formation of the blowholes is directly caused by the evaporation of the thin plated nickel of the surface of the battery case. The thin plated nickel is heated while the laser beam is melting the upper material (aluminum). Moreover, the boiling temperature of nickel and aluminum is similar. The nickel has low thermal conductivity, therefore, the heat loss due to heat conduction is negligible. As the result, the plated nickel is easy to be evaporated while the upper material (aluminum) is still in the liquid state. The nickel evaporation produces a high pressure which is so-called recoil pressure. This pressure subjects to the melt of the upper material and create the hole entrance. The laser welding process, characterizing by high cooling rate, makes the molten material be solidified easily. Consequently, the blowholes are formed after the rapid cooling process. At laser power of 10 W, the weld surface without the metal tube exhibits the formation of both blowholes and spatters as shown in Figure 6a. Moreover, the weld metal is detected to form in overlap configuration. This contributes to the formation of the weld bead with the bead width of 64.29 µm. In contrast, the surface of the weld using the metal tube shows a significant difference. More blowholes are produced in the region. Sometimes, these blowholes link together and generate a linear trench. It is noteworthy that spatters are founded progressively when the metal tube is applied. The measurement of the width of the weld bead with the use of the metal tube reports a width of 60.71 µm. The observation of the surface of the weld with and without the metal tube at the laser power of 15 W is shown in Figure 6b,e, respectively. Meanwhile, an increase of the weld metal in the weld without the metal tube is clearly observed. It is also interesting to note that, a reduction of the formation of the blowholes on both surfaces is obtained as the higher laser power is used. when the laser power of 20 W is used, both welds produce the messy surfaces caused by the growth of the weld metal as well as the generation of the spatter. A clear comparison of the spatter distribution on the surface between two welding configurations is shown in Figure 7. The spatter distributing on the surface of the weld with the metal tube is more than that without the metal tube. In addition, one can be obtained from the observation is the formation of the explosive holes at the intersection of the zigzag hatching line and circular line. The explosive hole is believed to be formed after the generation of the blowholes. As the blowholes are created during the circular path welding, massive power energy will be absorbed inside the blowholes at the intersection. This results in greater metal evaporation as well as the recoil pressure. Since a large amount of the melt evaporates quickly, the blowholes largely expand to become the explosive holes. The difference in the size of the explosive holes between two welding configurations is also recognized. Besides, after welding, a burn mask and spatter are found on the inside wall of the metal tube.

From the above result, it is clear that the use of the metal tube has both negative and positive contributions on the weld surface appearance. It is important to highlight the fact that the influence of the metal tube and the gas plume trapped inside the metal tube on the energy absorption of the material has a significant effect on the weld morphology. In particular, the number blowholes formed in the weld with and without the metal tube is different, which is mainly due to the difference of power energy absorption. It should be noted that the blowholes formation on the weld surface is different from the porosity formation inside the weld metal. The blowholes are formed on the weld surface which means the evaporation of the upper materials affects this formation. The higher laser power is used, the more laser energy is produced. Therefore, the laser beam can penetrate deeper into the material. Thus, more material is melted, and more material flows upwards in comparison with the lower laser power. The lower material flowing upwards can fill fully the blowholes. As the result, the blowholes are not recognized. In comparison with the welding using the metal tube, higher power energy is absorbed by the material when the metal tube is not applied. As a result, the less formation of the blowholes on the weld surface is observed on the weld surface. The reduction of the blowholes also happens when the laser power increases. This shows that the higher energy is absorbed, the higher recoil pressure is produced by the Ni evaporation. Together, the findings confirm the influence of the metal tube on the top surface morphology. Generally, the application of the metal tube has resulted in more weld defects such as spatters, and blowholes in the welding zone in comparison with the weld without the metal tube. In other words, a finer weld surface is produced without the use of the metal tube.

As analyzed above, the formation of the explosive hole and spatters in two welds are different. This reveals the advantage of using the metal tube. When the metal tube is used, it helps to gather the spatter inside it, hence the outside structure is protected from the spatter ejection. Moreover, the explosive hole is supposed to cause a bad surface appearance of the weld. Overall, the use of the metal tube results in smaller explosive holes in comparison without the metal tube.

**Figure 8:**
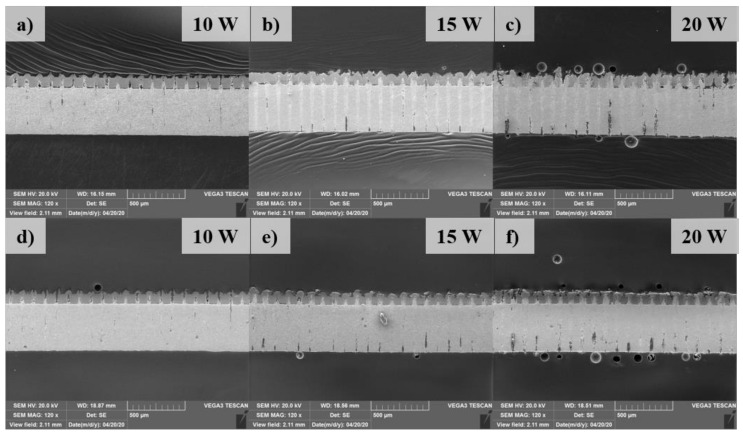
SEM images of the overview of the cross-section of the weld. (**a**–**c**): Weld without the metal tube; (**d**–**f**): Weld with the metal tube.

**Figure 9:**
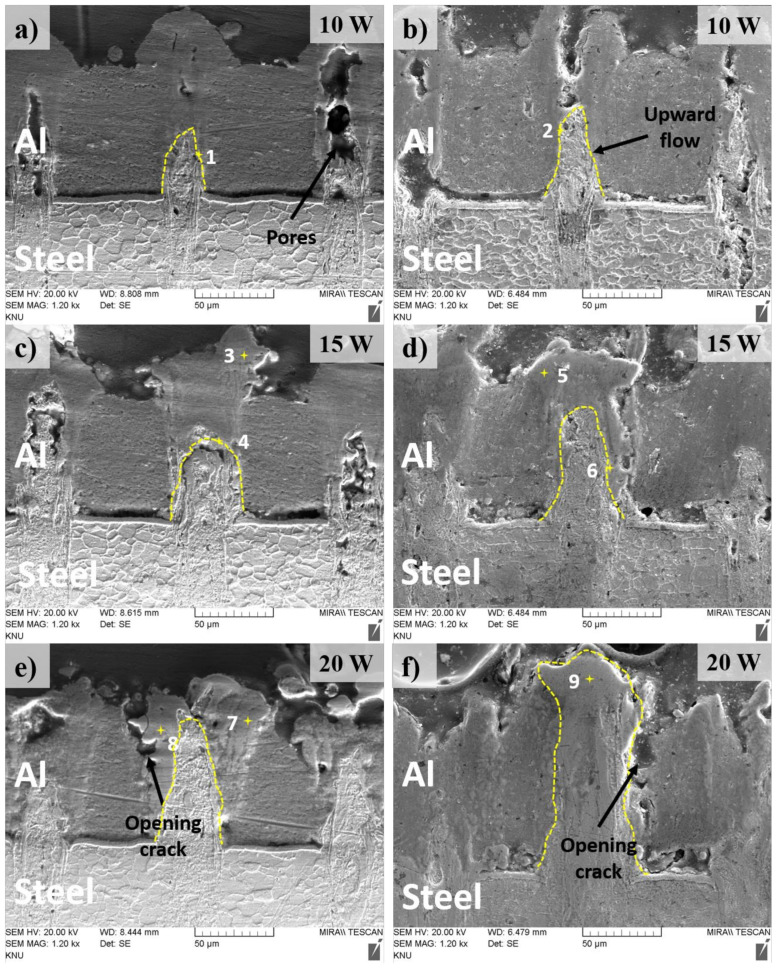
SEM images of the cross-section observation. (**a**), (**c**), (**e**): Weld using the metal tube; (**b**), (**d**), (**f**): Weld without the metal tube.

The cross-section of the weld is obtained by cutting perpendicularly with the hatching path in the middle of the welding zone. The cross-section is etched with the 4% Nital solution 4 mL ethanol (C_2_H_5_OH) + 100 mL nitric acid (HNO_3_). An overview of the cross-section view in the welds after etching is shown in Figure 8. A comparison of the high manification observation of the cross-section in the weld with and without the metal tube is shown in Figure 9. A novel finding is that the lower material is drawn to the upper material as shown in Figure 9. The phenomenon is suspected to be accelerated by the formation of the recoil pressure, as well as the characteristics of the present laser source. It should be noted that the recoil pressure is produced by metal evaporation, this pressure tends to flow away the remaining melt in the keyhole. Meanwhile, with the high-intensity laser source and focus laser spot, a high aspect-ratio weld (thin weld) is created. Therefore, the combined effect of the recoil pressure and the thin weld force the lower molten material flow upwards. The comparison of the cross-section between two welding configurations presents the difference in the amount of the lower material flowing upwards. The difference in the upward flow of the lower materials can be recognized by the height of the upwards penetration. Without the metal tube, it can be seen that the amount of the upward flow of the lower material is more than that with the metal tube. The decrease of the upward flow in the weld using the metal tube is due to the reduction of the laser energy absorbed by the material. The loss of laser energy occurs when it transmits through the trapped plume inside the metal tube. As the result, the less laser energy absorbed, the less upward penetration is produced.

As the substrate materials of Al and Fe have low solubility, the intermetallic compounds (IMCs) are formed during diffusion. The marked points in the Fusion Zone (FZ) indicate the supposed IMCs area as those either reveal a unique structure or locate at the interface between diffused materials. The Al and Fe composition of the marked points as well as the possible phases which are determined according to the Fe-Al phase diagram are shown in Table 1. It is found that the content of Ni is also detected at the interface, which agrees with the explanation for the formation of the blowholes as mentioned above. The result from EDX analysis shows that FeAl intermetallic is found at the interface of the Al and Fe at the laser power of 10 W in two welding configurations.

However, the Al-rich IMCs including FeAl_2_ and Fe_2_Al_5_ are obtained when both joints are welded with the laser power of 15 W. The intermetallic of Fe_2_Al_5_ is likely found at the upper part of the FZ as indicated at marked point 3 and 5. Interestingly, the weld without the metal tube produces α-Fe (ferrite) at the top part of the FZ at the laser power of 20 W, while Fe_2_Al_5_ is still produced in the same region in the weld using the metal tube. The observation at the laser power of 20 W also exhibits a significant difference in the FZ between two welds. When the metal tube is used, the FZ is observed with the solidified lower material surrounded by a thick IMCs layer. Meanwhile, the weld without the metal tube shows a thinner straight flow of the lower material which is supposedly due to high power energy absorption. Without the metal tube, the upward flow of the lower material partially inserts to the gap between the upper and lower material.

A further novel finding within the cross-section observation is that there is a gap between the upper and lower material in the weld without the metal tube, while the gap is minimized when the metal tube is used. This result now provides evidence of the positive effect of the application of the metal tube. Without the metal tube, the recoil pressure and the upward flow of the molten material subject to the tab and increases the contact gap between the tab and the battery case. Meanwhile, when the metal tube is placed over the tab, it minimizes the increase of the gap during the welding process. Besides, the cross-section observation also reveals the formation of pores and opening cracks within the weld. The pores are mostly found at the low laser power such as 10 W, while several opening cracks are formed at the lase power of 20 W in both welding configurations. The finding of the IMCs explains the formation of weld defects such as opening cracks since these IMCs are relatively brittle. As the thick IMCs layers are found in the weld with the laser power of 20 W, cracks are likely formed at this weld.

### 3.3. Hardness Distribution

**Figure 10:**
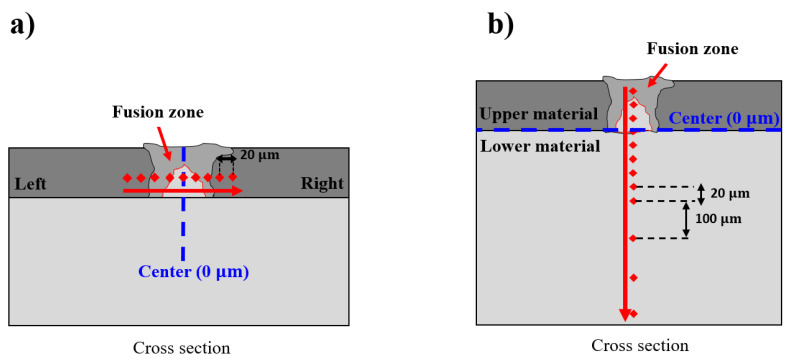
Schematics of the hardness measurement: (**a**) Horizontal direction, (**b**) Vertical direction.

In order to examine the effect of the IMCs on the hardness distribution on the fusion zone, the hardness measurement is conducted in both horizontal and vertical directions. Figure 10 exhibits the schematics of the hardness measurement of the welds in both horizontal and vertical directions at three different laser powers of 10 W, 15 W, and 20 W. In the horizontal direction, a total of 9 points around the FZ are measured. However, in the vertical direction, the measurement is performed along the fusion zone from the upper material to the lower material. There is a total of 12 measured points, as shown in Figure 10b.

**Figure 11:**
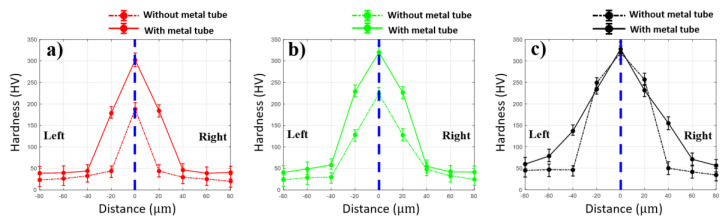
Hardness measurement in the horizontal direction with the laser power of (**a**) 10 W, (**b**) 15 W, and (**c**) 20 W.

The hardness profiles in the horizontal direction of the weld in both configurations are shown in Figure 11. The results in both welds have the same tendency of the hardness distribution. The hardness increases as the distance is close to the fusion zone and the maximum hardness is obtained in the middle of the fusion zone. At laser powers of 10 W and 15 W, the hardness remains under 60 HV outside the region of 40 µm from the fusion zone at both sides. Meanwhile, the weld without the metal tube shows higher hardness distribution than that with the metal tube. A significant difference between the two welding configurations is observed at 20 µm on both sides far away from the middle of the fusion zone. In particular, in the weld with 10 W, the hardness at the distance of 20 µm from the middle of the fusion zone is 178.6 HV and 184.3 HV on the left and right, respectively. In comparison, the hardness distribution at the region in the weld using the metal tube remains at 44.3 HV on the left and 44 HV on the right. However, when the laser power of 15 W is used, the hardness at 20 µm away from the middle of the fusion zone in the weld using the metal tube increases significantly. At the laser power of 20 W, the hardness at the distance of 20 µm from the middle of the FZ in both welds are mostly equal as shown in Figure 11c. The difference of the hardness profile of the weld with and without the metal tube is explainable with the amount of the mixing of the upper and lower materials as well as the IMCs. Under the effect of the plume in the weld using the metal tube, less laser energy is absorbed by the materials. This results in less mixing of the upper and lower materials as well as less upward penetration of the lower material. It should be noted that the lower material (steel) has higher hardness. Besides, the hardness significantly increases when the metal is melted and solidified, explained by the steel quenching effect [32]. Moreover, a large amount of molten material is solidified in the FZ. As a result, the high hardness is obtained at the center of the FZ and the hardness distribution in the weld without the metal tube is generally higher than the other.

Besides, the hardness distribution in both weld configurations reveals a promising finding of the effect of the plated Ni. The typical hardness value of Fe–Al IMCs are as follows: Fe_2_Al_5_-1013 HV, FeAl-470 HV, FeAl_2_-1060 HV [33]. However, the maximum hardness obtained in this measurement is 327.6 HV. It is evident that the currently reported hardness is lower than the typical hardness of the IMCs. Moreover, the Ni is also found in the region of the IMCs. Therefore, it can be assumed that the Ni joining in the Fe–Al IMCs slightly reduces the hardness of the IMCs. The comparison of the hardness between two welding configurations at a laser power of 20 W shows an unfamiliar result. The most reliable explanation for this comparison is the formation of a thick IMCs layer around the lower material which penetrates the upper material as shown in Figure 9e.

**Figure 12:**
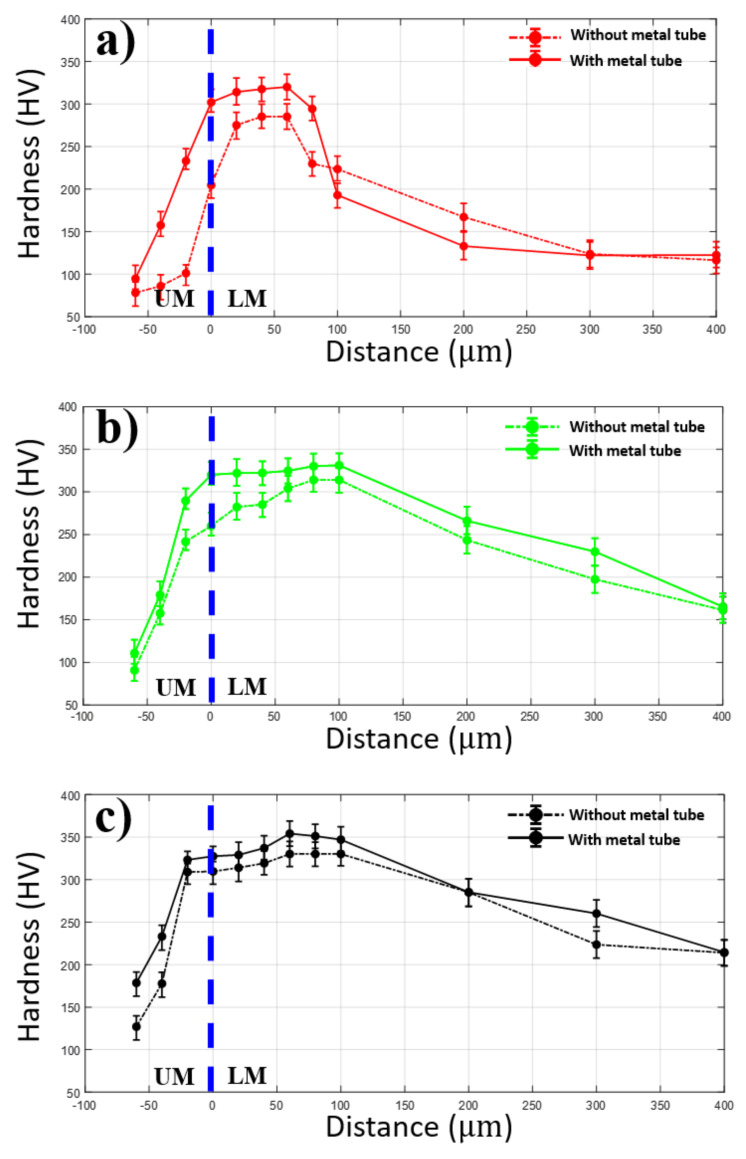
Hardness following the vertical direction. (**a**) 10 W, (**b**) 15 W, (**c**) 20 W.

The hardness measurement in the vertical direction of two welding configurations is shown in Figure 12. As shown in the graphs, the weld using the metal tube tends to produce an average lower hardness profile than the weld without the metal tube. As all the measured points are in the order from the upper material (UM) to lower material (LM), the hardness distribution firstly increases when the distance is close to the interface of the upper and lower material. Within the lower material, the hardness continuously rises to reach a maximum value at the distance of 100 µm from the interface, and then the hardness decreases gradually as the distance is far from the interface. However, in each laser power, there is a remarkable difference in hardness distribution between two weld configurations. At laser power of 10 W, the maximum hardness of the weld without the metal tube at this laser power is 320.1 HV, while the maximum hardness of 285.2 HV is obtained at the given laser power when the metal tube is applied. At laser power of 15 W, the hardness distribution in the weld without the metal tube remains consistently higher than that in the weld using the metal tube. At the distance of 100 µm from the interface of the upper and lower material, both the maximum hardness of the weld with and without the metal tube are obtained, which are 314.1 HV and 331.2 HV, respectively. When the joint is welded with 20 W, the similar hardness distribution to the other welds along the direction can be obtained. The hardness increases sharply when the distance is close to the interface, the peak hardness in both weld configurations is observed at 60 µm away from the interface in the lower material as shown in Figure 12c. The lower hardness distribution of the weld using the metal tube is indirectly caused by the lower absorbed power energy affected by the plume and Gaussian beam distribution. The lower energy absorption correlates to a shallower penetration of the laser beam into the lower material in the weld using the metal tube in comparison to the weld without the metal tube. This leads to less material melted and solidified. As a result, the hardness distribution is slightly lower with the use of the metal tube.

### 3.4. Mechanical Strength

**Figure 13:**
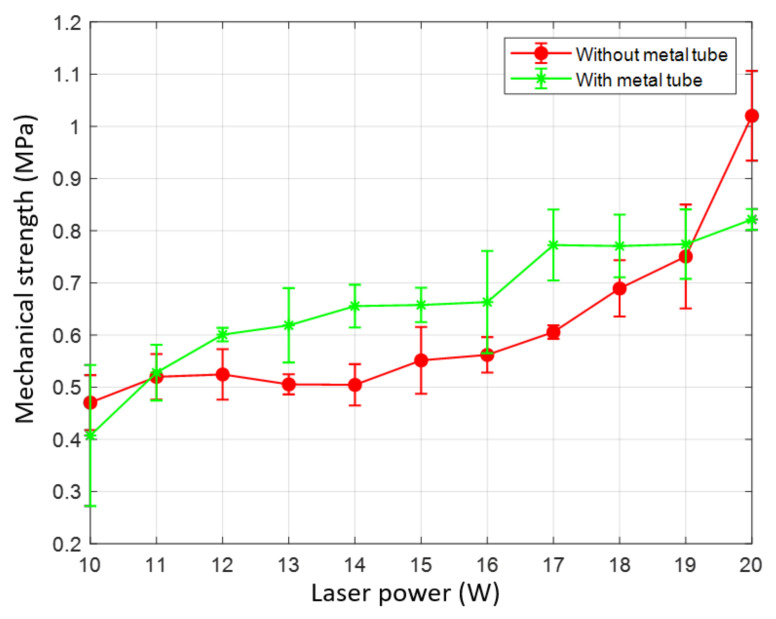
Tensile strength between the weld with and without the metal tube.

The mechanical strength profile in both welding configuration is shown in Figure 13. The peel test is performed with the speed of 1 mm/s until the battery case and the tab are completely detached. The result shows that the failure during the test occurs at the boundary of the welding zone. The maximum stress is recorded and exhibited in Figure 13. As shown in the graph, the tendency of increasing strength at the higher laser power is obtained at both welds with and without the metal tube. Nevertheless, there is a considerable difference in the mechanical strength between the two welding configurations. Overall, the lowest strength is obtained at laser power of 10 W in both welds. In particular, the average strength at laser power of 10 W in the weld without the metal tube is 0.47 MPa. The average strength of 0.41 MPa is obtained at laser power of 10 W in the weld using the metal tube. However, the use of the metal tube results in the higher strength at the laser power within the laser power range of 11–19. There is a significant difference in the average strengths at 20 W in the weld with and without the metal tube, which are 0.82 MPa and 1.02 MPa, respectively. 

It can be seen that the mechanical strength of the weld is changed when the metal tube is used. The average lower tensile strength of the weld without the metal tube is due to the contact gap between the upper and the lower material in the weld. This gap makes the tab and the battery case easy to be detached. Nevertheless, the opposite result is obtained at laser powers of 10 W and 20 W. As discussed, the low strength of the weld using the metal tube at the laser power of 10 W is due to the increase of the formation of the blowholes on the weld surface and pores in the weld seam. On the other hand, the formation of the IMCs layer mentioned above is suspected of the decrease of the tensile strength of the weld at the laser power of 20 W. In summary, the use of the metal tube generally strengthens the weld by reducing the gap in the middle of the battery case and the tab, while the exception is caused by the weld defects and the IMCs.

### 3.5. Electrical Resistance

**Figure 14:**
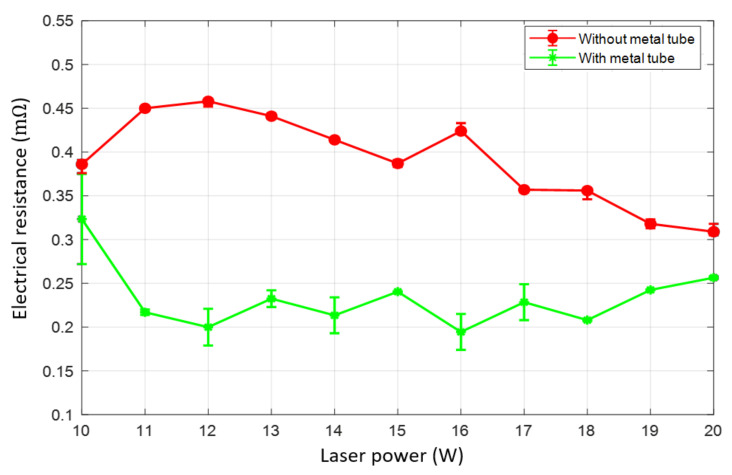
The electrical resistance of the weld with and without the metal tube.

The electrical resistance of the weld is measured with the four-wire method. The measurement results of the weld with and without the metal tube are compared in Figure 14. Overall, the weld using the metal tube produces lower electrical resistance in comparison with the weld without the metal tube. As shown in the graph, the weld without the metal tube produces the electrical resistance in the interval of 0.31 mΩ to 0.46 mΩ. In contrast, a reduction of the electrical resistance of the weld using the metal tube is obtained when laser powers of 10 W, 11 W, and 12 W are used. However, the electrical resistance varies in the interval of 0.174 mΩ and 0.258 mΩ as the laser power increases in the range of 13 W and 20 W.

It should be noted that minimizing the gap between the specimens significantly increases the electrical conductivity. This explains the lower electrical resistance when using the metal tube. Moreover, an interesting finding is a relation between the mechanical strength and the electrical resistance of the weld. The higher strength in the welding configuration with the metal tube corresponds to the lower electrical resistance. The increment of the electrical resistance at laser powers of 10 W and 20 W correlates with the degrading of the mechanical strength of the weld.

## 4. Conclusions

As the welding of the battery case and the tab in the cylindrical lithium-ion battery is performed at the bottom of the battery case, it is hard to keep contact between the battery case and the table. Moreover, the ejection of the spatter from the welding zone damages the jellyroll because the welding zone is surrounded by the jellyroll. Therefore, a novel idea of using a metal tube inserted in the middle of the jellyroll is proposed to solve these issues. However, the use of the metal tube also causes some changes such as trapped plume inside the metal tube, and Gaussian distribution of the laser beam. In the present experiment, the application of the metal tube assisting laser welding of the battery case and the tab is attempted. The effect of the metal tube in the weld on the morphology, mechanical, and electrical properties of the weld are studied. According to the analysis, several considerations are concluded as follows:

- The use of the metal tube results in trapped plume inside the metal tube, which has a significant effect on the change of the weld morphology and properties.
- When the metal tube is used, the weld surface exhibits more spatters distributed in the welding zone. At the low laser power (10 W), the weld surface using the metal tube reveals more blowholes, while the size of the explosive holes is smaller in comparison with the weld without the metal tube.
- The improved hardness distribution together with the tensile strength of the weld is improved by the use of the metal tube (i.e., clamping effect of the metal tube).
- The application of the metal tube reduces the contact gap between the battery case and the table. Hence, this positive contribution significantly increases the electrical conductivity of the weld.

Though such positive effects have been recognized with the application of the metal tube, it is important to designate the optimum laser parameters which provide the greatest weld in combination with the use of the metal tube. Moreover, the influence of different dimensions of the metal tube should be further investigated.

## Figures and Tables

**Table 1:** EDS result of the marked points in the Fusion Zone (FZ) in the weld.

Point	Al (at.%)	Fe (at.%)	Ni (at.%)	Possible Phase
1	51.02	11.45	13.17	FeAl
2	54.38	17.45	5.49	FeAl
3	74.63	9.63	0.43	FeAl_3_
4	66.17	9.11	2.71	FeAl_2_
5	49.03	16.95	0.48	FeAl
6	59.27	10.78	0.95	Fe_2_Al_5_
7	57.83	15.72	2.65	Fe_2_Al_5_
8	56.20	16.04	1.95	Fe_2_Al_5_
9	17.8	47.55	0	α-Fe
